# Safety and efficacy of a novel three-dimensional printed microporous titanium prosthesis for total wrist arthroplasty in the treatment of end-stage wrist arthritis

**DOI:** 10.3389/fbioe.2022.1119720

**Published:** 2023-01-10

**Authors:** Xingbo Cai, Bin Wang, Bihuan Zhang, Yue Zhang, Teng Wang, Wei Lin, Youjun Huang, Bingquan Chen, Siyuan Zhou, Sheng Lu, Yongqing Xu

**Affiliations:** ^1^ Graduate School, Kunming Medical University, Kunming, China; ^2^ Department of Orthopedics, 920th Hospital of Joint Logistics Support Force, PLA, Kunming, China; ^3^ Suzhou MicroPort OrthoRecon Co., Suzhou, China; ^4^ Department of Orthopedics, The First People’s Hospital of Yunnan Province, Kunming, China

**Keywords:** 3D-printed microporous titanium artificial wrist joint, artificial wrist joint prosthesis, end-stage carpal arthritis, long-term follow up, total wrist arthroplasty

## Abstract

**Background:** Total wrist arthroplasty is an effective treatment for end-stage wrist arthritis from all causes. However, wrist prostheses are still prone to complications such as prosthesis loosening and periprosthetic fractures after total wrist arthroplasty. This may be due to the wrist prosthesis imprecise matching with patient’s bone. In this study, we designed and developed a personalized three-dimensional printed microporous titanium artificial wrist prosthesis (3DMT-Wrist) for the treatment of end-stage wrist joint, and investigated its safety and effectiveness.

**Methods:** Total wrist arthroplasty was performed using 3DMT-Wrist in 14 cases of arthritis between February 2019 and December 2021. Preoperative and postoperative visual analog scale scores, QuickDASH scores, wrist range of motion, and wrist grip strength were evaluated. Data were statistically analyzed using the paired samples *t*-test.

**Results:** After 19.7 ± 10.7 months of follow-up, visual analog scale decreased from 66.3 ± 8.9 to 6.7 ± 4.4, QuickDASH scores decreased from 47.4 ± 7.3 to 28.2 ± 7.6, grip strength increased from 5.6 ± 1.4 to 17.0 ± 3.3 kg. The range of motion improved significantly in palmar flexion (30.1° ± 4.9° to 44.9° ± 6.5°), dorsal extension (15.7° ± 3.9° to 25.8° ± 3.3°), ulnar deviation (12.2° ± 3.9° to 20.2° ± 4.3°) and radial deviation (8.2° ± 2.3° to 16.2 ± 3.1). No dislocation or loosening of the prosthetic wrist joint was observed.

**Conclusion:** Total wrist arthroplasty using 3DMT-Wrist is a safe and effective new treatment for various types of end-stage wrist arthritis; it offers excellent pain relief and maintains the range of motion.

## 1 Introduction

Total wrist arthroplasty (TWA) has been performed for more than 40 years, and has offered good results in terms of pain relief and improvement of range of motion (ROM) and wrist function; however, the carpal component tends to loosen and subside on long-term follow-up. The first-generation Swanson silastic wrist prosthesis was developed in 1967, and offers the longest available follow-up data for over 15 years; however, silicone is prone to fracture and this procedure has a revision rate of 15%–41% (Damert, 2019). In the 1970s, the second generation of wrist prosthesis was developed by Meuli (Sulzer, acquired by Zimmer) and Volz (Howmedica, acquired by Stryker). These titanium prostheses had a bifurcated design and required bone cement for fixation. Unfortunately, it was demonstrated a high failure rate (Srnec et al., 2018). The third generation of artificial wrist prostheses were developed in 2000; these were mainly biaxial artificial wrist joints, biaxial total wrist implants (DePuy), and universal total wrist implants (KMI). These implants had a prosthetic coating, polyethylene components, and involved screw fixation; however, the main components still required bone cement fixation (Berber et al., 2018; Elbuluk et al., 2018; Reigstad and Røkkum, 2018; Srnec et al., 2018). The fourth generation prosthetic wrist joints emerged in 2003. These included the ReMotion (Stryker) and Maestro (Zimmer Biomet) systems, and involved biologic fixation without the need of cement (Herzberg et al., 2012; Bidwai et al., 2013; Boeckstyns et al., 2013; Sagerfors et al., 2015). This represented a significant improvement over previous generation. In this context, studies have reported better results with the fourth generation of artificial wrist joints (Bidwai et al., 2013; Froschauer et al., 2021). In contrast to wrist fusion, TWA preserves wrist mobility and provides effective pain relief (Fischer et al., 2020; Berber et al., 2020; Matsui et al., 2020; Zhu et al., 2021). However, the carpal component still tends to loosen on long-term follow-up (Reigstad et al., 2017).

Additive manufacturing, often known as three-dimensional (3D) printing or prototyping, can convert 3D digital models into functioning components regardless of their geometry (Raheem et al., 2021). Selective laser melting metal 3D printing technology is able to construct complex free-form surfaces, print parts with proper toughness and elastic modulus, and even surface coating layer (Yang et al., 2021). Owing to these properties, 3D printing technology has been employed in the field of hand surgery research for artificial lunar bone replacement and good results have been obtained in the treatment of advanced Kienböck’s disease (Ma et al., 2020). Trabecular bone grows within the grid of these implants and permeates throughout the porous structure. In addition, the body identifies human bones and microporous titanium as one structure, leading to good osteogenesis (Núñez et al., 2013). In the field of TWA research, customized artificial wrist prostheses have not been made using 3D-printed personalized microporous titanium. This study therefore aimed to investigate the safety and feasibility of developing a personalized 3D-printed microporous titanium artificial wrist prosthesis (3DMT-Wrist) system for the treatment of end-stage wrist arthritis.

## 2 Materials and methods

### 2.1 Case selection criteria

Patients fulfilling the following criteria were included in this study: 1) diagnosed with rheumatoid arthritis, osteoarthritis, or deformity of the wrist with significant pain and limitation of movement (or combined with ulnar displacement instability of the radial wrist joint); 2) requiring total wrist fusion (including fusion of the radial wrist and midcarpal joints) with low requirements for life quality; 3) having severe ischemic necrosis of the wrist bones with an advanced stage of joint collapse; and 4) having good general condition, with good function of the heart, liver, kidney, brain, and other organs.

The following patients were excluded: 1) those with poor general condition and serious complications, 2) having *tuberculosis* or septic infection of the wrist joint, and 3) those demonstrating poor compliance or the inability to perform relevant postoperative functional rehabilitation exercises.

### 2.2 General information

This study included 14 patients who underwent TWA using the individualized 3DMT-Wrist system at our hospital between February 2019 and December 2021 (Table 1). They were seven men and seven women in the study; they had an average age of 45.2 ± 13.5 (23–70) years. Overall, 3, 1, and 10 cases had rheumatoid arthritis, an open segmental defect caused by gunshot injury, and osteoarthritis, respectively. Patients were evaluated both preoperatively and postoperatively, based on findings on wrist radiography and computed tomography, pain levels, and wrist function. The level of wrist pain was evaluated based on the visual analog scale (VAS) scores and wrist function, which was evaluated based on the QuickDASH score, wrist grip strength, and wrist ROM (palmar flexion, dorsal extension, ulnar and radial deviation, pronation, and supination). Grip strength measurements were performed by a resident surgeon using the JAMAR hydraulic hand dynamometer (Sammons Preston, United States); the ROM was recorded using a goniometer. Radiographs were obtained at 1, 3, 6, and 12 months postoperatively. At least 2 mm of movement around the prosthesis-bone interface on serial radiographs were considered to be indicative of implant loosening; two surgeons performed the examination consistently.

**TABLE 1:** General information (*n* = 14).

Characteristic		No.	%
Sex	Female	7	50.0
	Male	7	50.0
Disease Type	Osteoarthritis	10	71.4
	Traumatic arthritis	1	7.1
	Rheumatoid arthritis	3	21.4
Follow-up (month)	19.7 ± 10.7		
Age (years)	45.2 ± 13.5 (23–70)		

### 2.3 Design and research of the individualized microporous titanium artificial wrist joint

**FIGURE 1:**
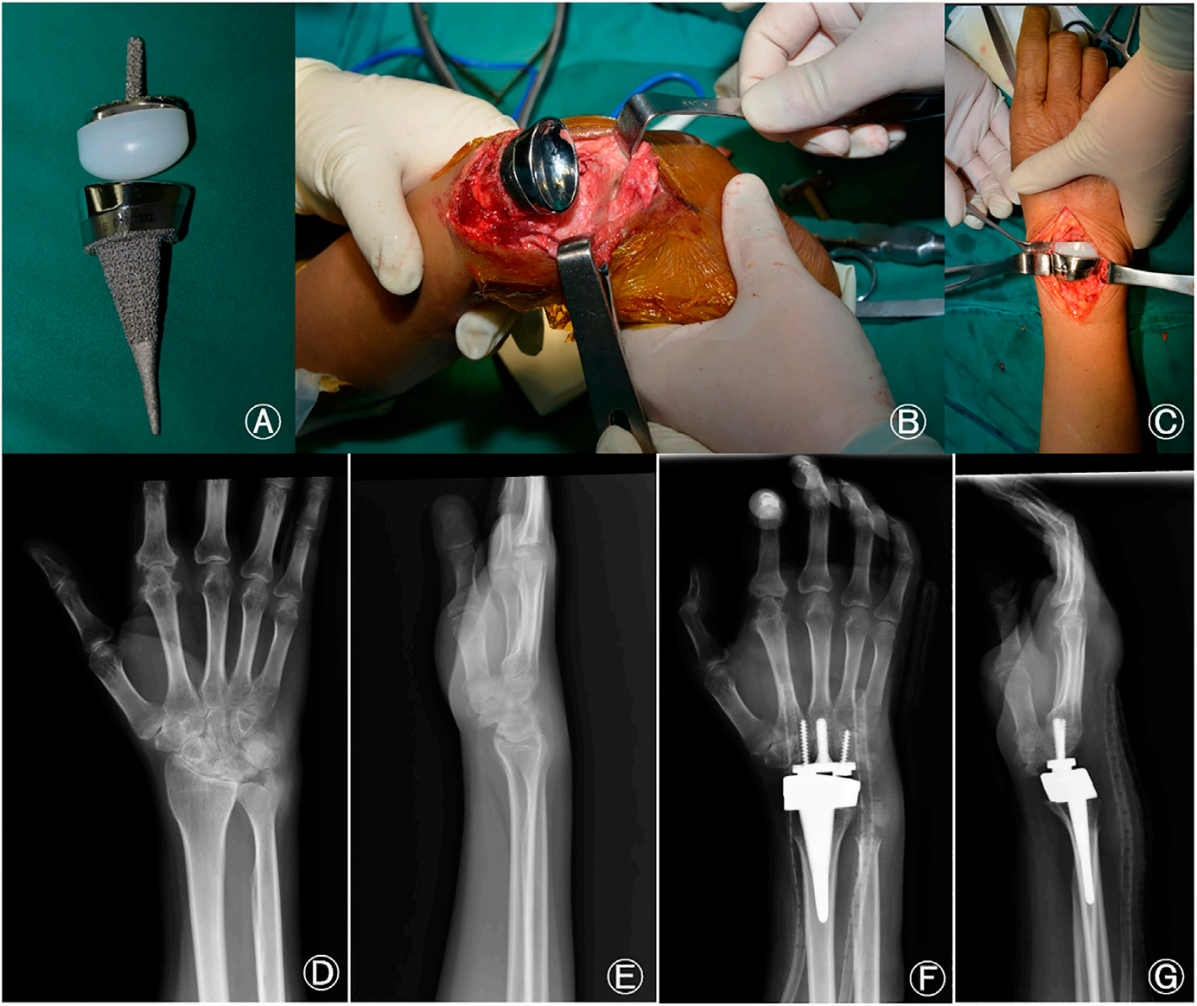
**(A)** Microporous titanium total wrist joint prosthesis, **(B)** Intraoperative carpal and radial osteotomy have been completed and the radial component is being installed. **(C)** Polyethylene has been inserted into the carpal component and the artificial wrist joint has been reset, **(D)** Preoperative posteroanterior radiographs of the wrist, **(E)** Preoperative lateral radiographs of the wrist, **(F)** Posteroanterior radiographs of the wrist at 1 month after TWA, **(G)** Lateral radiographs at 1 month after TWA. TWA, total wrist arthroplasty.

The 3DMT-Wrist was developed by our research team in collaboration with MicroPort OrthoRecon Co. As shown in Figure 1A, the microporous titanium artificial wrist joint consisted of three parts: carpal and radial components and a polyethylene joint ball. The 3DMT-Wrist body material is Ti6Al4V and was manufactured by a metal 3D printer (SLMS310, Xi’an Plastica Additive Technology Co., China). The joint ball material is highly cross-linked ultra-high molecular weight polyethylene, which is manufactured by mechanical processing. The carpal component included a carpal platform, a central carpal column, screws on the left and right sides, a cylindrical projection on the carpal platform, and a carpal stem of Ti6Al4V with a diameter of 5 mm (which was fixed by inserting into the waist of the capitate); it had a 1-mm-thick porous surface to facilitate bone growth. Screws with a diameter of 4.0 mm could be placed in the screw holes on both sides of the carpal stem; in order to achieve fixation of the carpal component, radial and ulnar screws were aimed at the second and fourth metacarpal, respectively.

The polyethylene joint ball had a semi-ellipsoidal shape and was 40 mm long and 18 mm wide. It was prepared in three different sizes based on the height; the sizes were as follows: 12.5 mm (size I), 17.5 mm (size II), and 22.5 mm (size III). The polyethylene joint ball formed an elliptical joint with the radial component to maximize restoration of the wrist joint motion pattern.

The radial component consisted of a radial shank and an articular socket composed of Ti6Al4V and a CoCrMo alloys, respectively. The length of the radial shank was 65 mm and the proximal diameter was either 5 mm (size S#) or 7 mm (size L#); the ulnar deviation angle was approximately 15°. The surface of the radial stalk was porous and had a thickness of 1 mm to facilitate bone growth; the articular fossa was polished to reduce friction between the articular surfaces.

**FIGURE 2:**
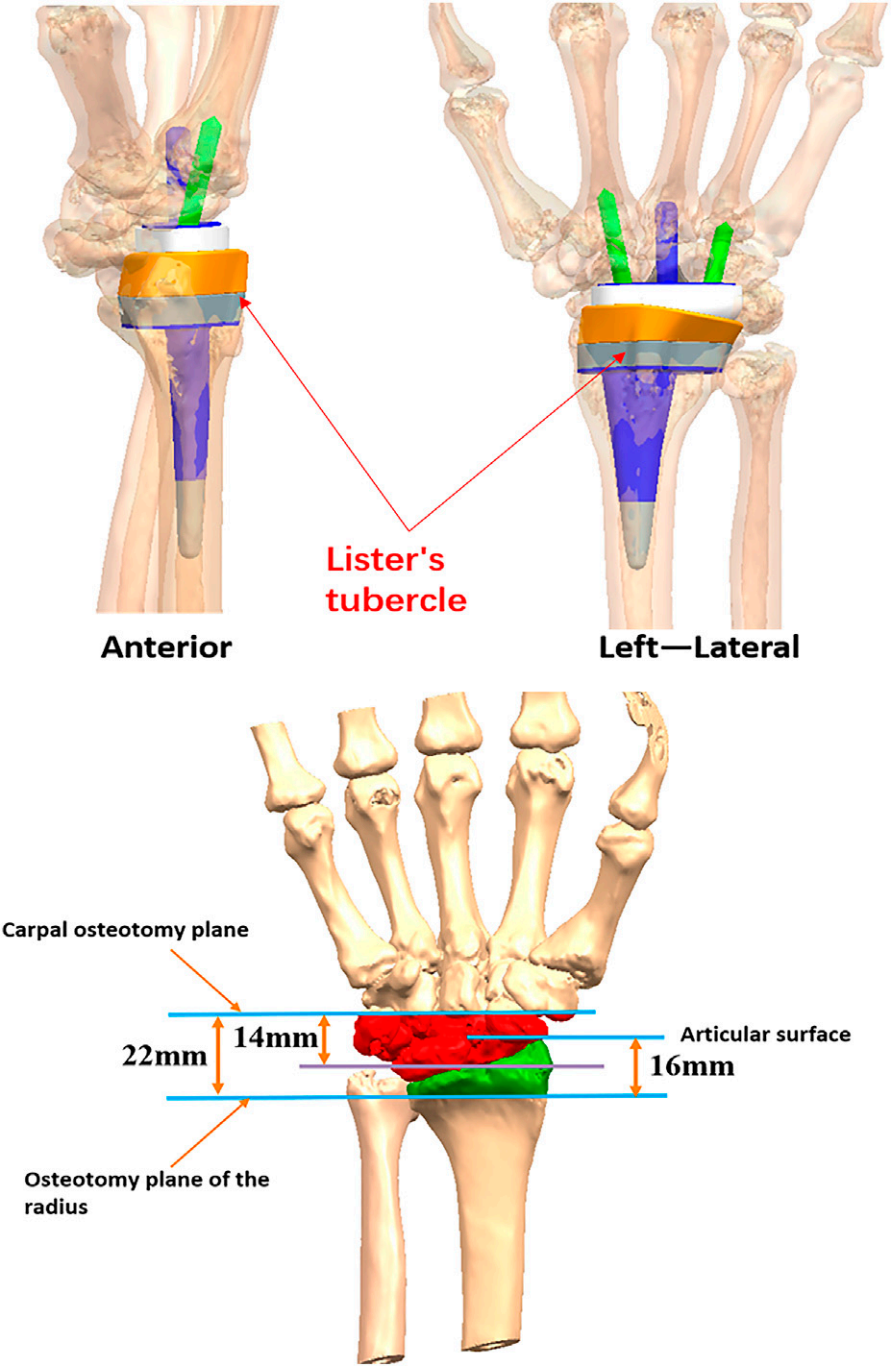
Preoperative wrist joint prosthesis design.

For clinical application, bilateral wrist joints, distal forearms, and metacarpals were first scanned by computed tomography; anatomical data relating to the wrist joints were obtained by mirroring the flip image. These data were provided to the company (which qualified based on biomechanical testing results) and the hospitals for use (Figure 2).

### 2.4 Surgical method

The affected limb was exsanguinated using a tourniquet after general or brachial plexus anesthesia; the tourniquet pressure was then maintained during surgery. An 8-cm incision was placed from the root of the third metacarpal to the distal radius *via* the Lister’s tuberosity; the skin and subcutaneous tissue were incised. The dorsal carpal capsule was lifted from the distal radius to the base of the metacarpal and the ulnar head was removed using biting forceps. The wrist was palmar flexed and the osteotomy line was marked on the distal radius using an electric knife; the median nerve was then protected using a periosteal stripper. The distal radius was osteotomized with a pendulum saw and reamed based on the individual medullary contour; as shown in Figure 1B, this was followed by insertion of the radial component. The thickness of the artificial carpal joint polyethylene and carpal component was measured to determine the thickness of the carpal bone to be osteotomized; after marking, the required segment was removed using a pendulum saw. The medullary cavity of the third metacarpal was then drilled from the center of the capitate section using an electric drill and the position was confirmed *via* radiographic fluoroscopy. The carpal stem was then driven in after reaming and fixed using screws on both sides. The radial stem was inserted, followed by insertion of polyethylene into the carpal component; as shown in Figure 1C, the artificial wrist joint was reset and moved to observe the ROM and check for the likelihood of dislocation. The procedure was confirmed to be satisfactory after fluoroscopic inspection of the artificial wrist joint. The tourniquet was then released, hemostasis was ensured, and the wound was irrigated. The *in-situ* suture of the U-shaped tissue flap was handled with care during the procedure to prevent dorsal dislocation of the wrist joint. The operation was completed after suturing of the incision and application of vacuum sealing drainage over the incision site.

### 2.5 Postoperative management

The cast was removed after 2 weeks and active and passive exercises were performed; the degree of dorsal extension was increased by 3–5° every 3 days to gradually increase the ROM. Cefazolin (1 g) was administered intravenously after surgery along with Aescuven Forte, which was administered orally. In this context, postoperative edema may occur easily after surgery to the wrist, as the soft tissue layer on the dorsal aspect is thin (with only tendons and no muscle coverage).

### 2.6 Postoperative imaging evaluation

Radiographs were obtained at 1, 3, 6, and 12 months after surgery.

### 2.7 Statistical analysis

The SPSS version 19.0 statistical software package was used for data analysis. Measurement data, including the VAS and QuickDASH scores, grip strength, and wrist mobility, were evaluated based on the ROM. Preoperative and postoperative data were compared using the paired *t*-test; *p* < .05 was considered statistically significant. All data have been presented as means with standard deviation.

### 2.8 Study design

Our study had a before–after design, in which the patients’ VAS and QuickDASH scores were assessed based on questionnaires administered before and after surgery. Data pertaining to wrist ROM and grip strength were obtained by physical measurement. Allocation concealment was not performed; blinding of treatment allocation was not possible owing to the nature of the interventions. This study was approved by the local ethics committee before patient enrolment.

## 3 Results

**FIGURE 3:**
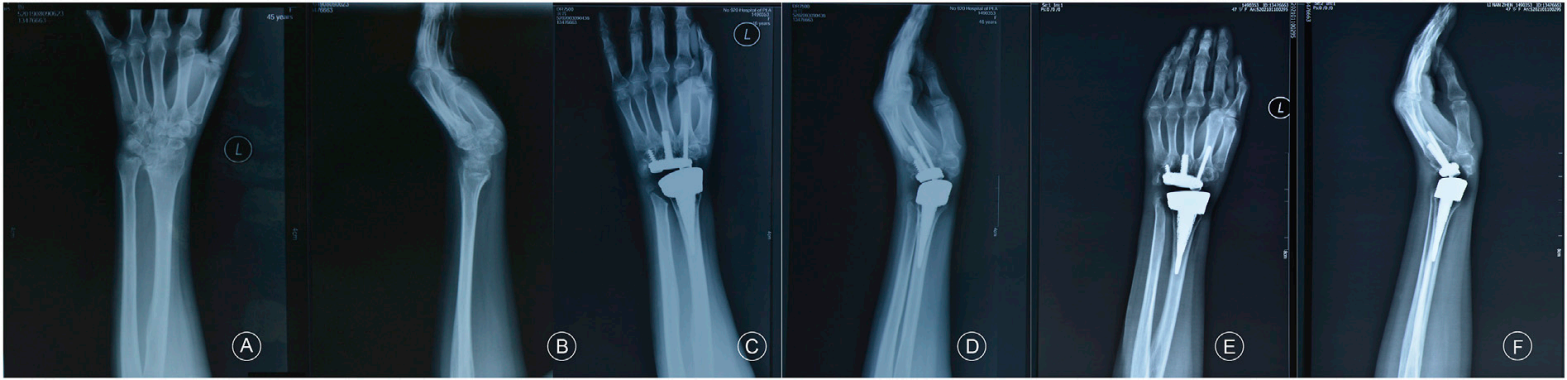
Osteoarthritis of the left wrist joint in a female patient aged 46 years. **(A,B)**: preoperative anteroposterior and lateral radiographs, **(C,D)**: postoperative anteroposterior and lateral radiographs at 6 months, **(E,F)**: postoperative anteroposterior and lateral radiographs at 12 months.

**FIGURE 4:**
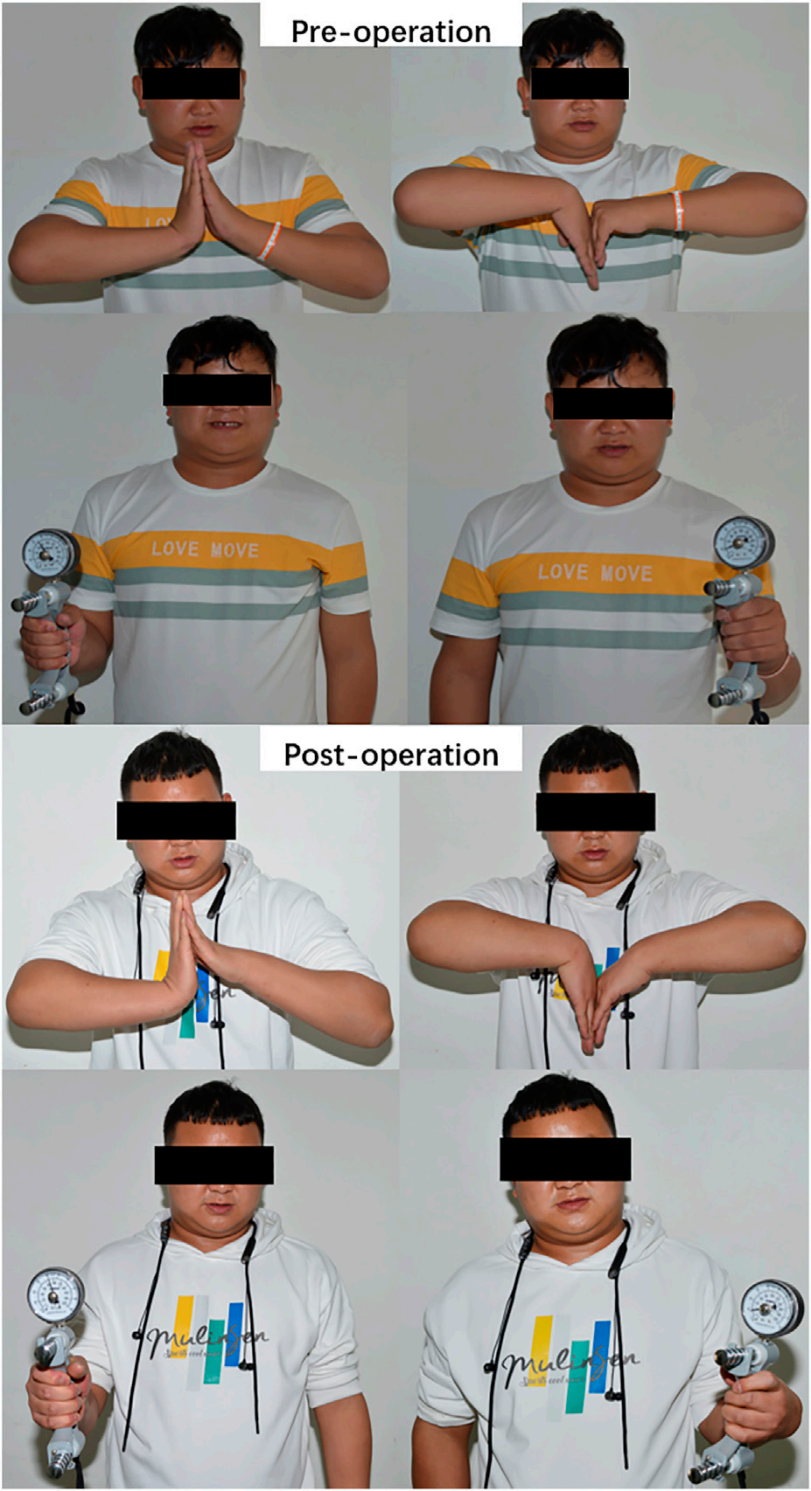
A patient with rheumatoid wrist arthritis with 24 months of follow-up after 3DMT-Wrist TWA.

**FIGURE 5:**
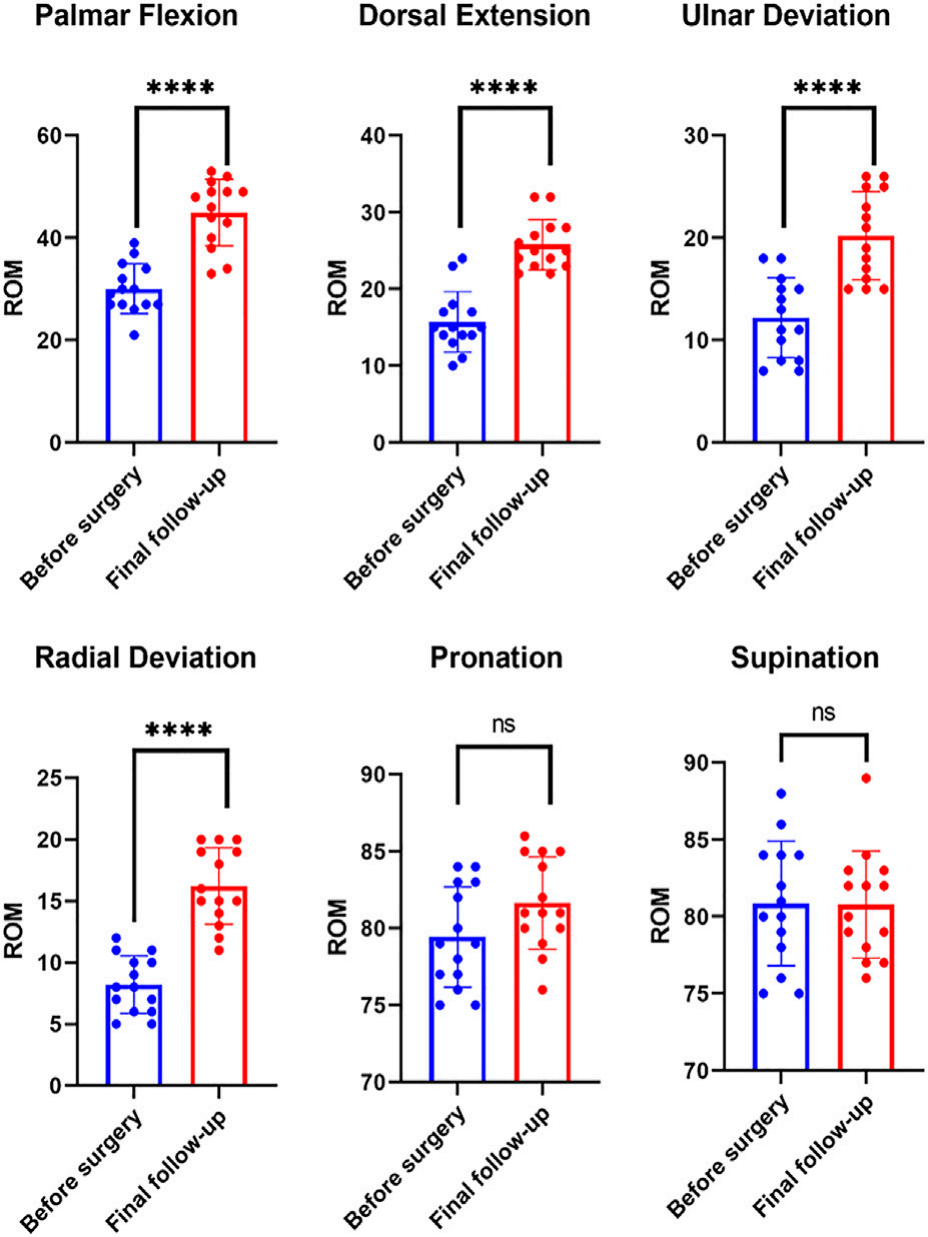
Comparison of ROM observed before surgery and at final follow-up. ROM, range of motion.

**FIGURE 6:**
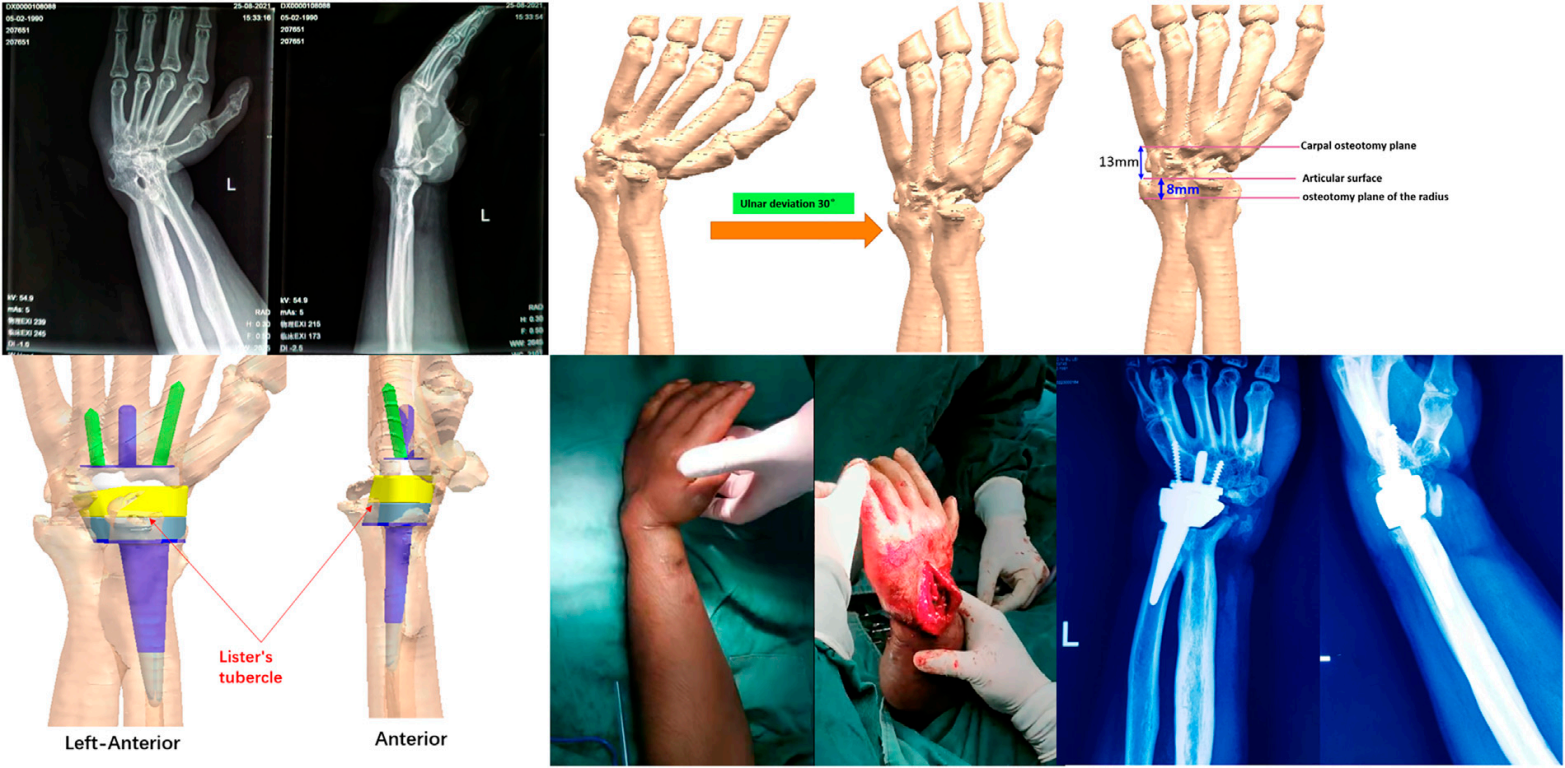
A complicated case with non-development of the carpal bones owing to a donkey bite at the age of 3 years. The patient demonstrated loss of wrist and forearm movement, but experienced pain relief and could move the wrist following personalized total wrist arthroplasty.

The mean age of the patients was 45.2 ± 13.5 (23–70) years, and the cohort had a male-to-female ratio of 1:1. Among the 14 patients, 10, 3, and 1 were diagnosed with osteoarthritis, rheumatoid arthritis, and traumatic arthritis, respectively. None experienced prosthesis loosening or dislocation or required revision surgery during the study period. One patient with a history of a gunshot wound in the wrist experienced occasional postoperative wrist and back pain due to severe trauma and surrounding soft tissue injury. All patients were followed up for 19.7 ± 10.7 (7–41) months (Figures 3, 4). At the final follow-up (Table 2), the VAS score decreased from 66.3 ± 8.9 to 6.7 ± 4.4 (t = 19.71, *p* < 0.05), and the QuickDASH score decreased from 47.4 ± 7.3 to 28.2 ± 7.6 (t = 16.18, *p* < 0.05). The grip strength increased from 5.6 ± 1.4 kg to 17.0 ± 3.3 kg (t = −22.0, *p* < 0.05) and the ROM improved significantly for palmar flexion, dorsal extension, and ulnar and radial deviation, with no significant differences for pronation or supination. The palmar flexion angle improved from 30.1° ± 4.9° to 44.9° ± 6.5° (t = −8.4, *p* < 0.05), the dorsal extension angle improved from 15.7° ± 3.9° to 25.8° ± 3.3° (t = −11.9, *p* < 0.05), the ulnar deviation angle increased from 12.2° ± 3.9° to 20.2° ± 4.3° (t = −4.9, *p* < 0.05), and the radial deviation angle increased from 8.2° ± 2.3° to 16.2° ± 3.1° (t = −14.2, *p* < 0.05). There was no statistical difference for pronation and supination (*p* > 0.05) (Table 3; Figure 5). Evaluation at the final follow-up showed no loosening or dislocation of the wrist in any patient. The preoperative and postoperative radiographs are shown in Figures 1D–G, 6.

**TABLE 2:** Comparison of VAS score, quick dash score, grip strength before surgery and final follow-up^a^.

Measurement	Before surgery	Final follow-up	t	*p*-value
VAS Score	66.3 ± 8.9	6.7 ± 4.4	19.71	<0.001
Quick DASH Score	47.4 ± 7.3	28.2 ± 7.6	16.18	<0.001
Grip Strength (kg)	5.6 ± 1.4	17.0 ± 3.3	−22.0	<0.001

^a^ Data are shown as mean ± SD. Comparison between preoperative condition and final flow-up.

**TABLE 3:** Comparison of ROM before surgery and final follow-up^a^.

Activity direction	Before surgery	Final follow-up	t	*p*-value
Palmar Flexion	30.1 ± 4.9	44.9 ± 6.5	−8.4	<0.001
Dorsal Extension	15.7 ± 3.9	25.8 ± 3.3	−11.9	<0.032
UInar Deviation	12.2 ± 3.9	20.2 ± 4.3	−4.9	<0.001
Radial Deviation	8.2 ± 2.3	16.2 ± 3.1	−14.2	<0.001
Pronation	79.4 ± 3.3	81.6 ± 3.0	−1.7	0.099
Supination	80.9 ± 4.0	80.8 ± 3.5	0.048	0.964

^a^ Data are shown as mean ± SD. Comparison between preoperative condition and final flow-up.

## 4 Discussion

In this study, the 14 participants who had undergone TWA with the 3DMT-Wrist system showed a decrease in VAS scores from 66.3 ± 8.9 to 6.7 ± 4.4 at 1.6 years after surgery; the QuickDASH scores also decreased from 47.4 ± 7.3 to 28.2 ± 7.6. These findings suggest that TWA using the 3DMT-Wrist system offered significant pain relief and improved function and ROM of the wrist joint. Except for in one patient with a history of a gunshot wound to the wrist (who had occasional dorsal wrist pain after surgery due to severe trauma and surrounding soft tissue damage), wrist pain had disappeared in all cases. The grip strength increased from 5.6 ± 1.4 kg to 17.0 ± 3.3 kg, probably reflecting the reduction in wrist pain; loosening of the prosthesis or joint dislocation were not observed on the radiographs. It is worth noting that all patients who underwent TWA using the 3DMT-Wrist system demonstrated excellent pronation and supination ability; this was mostly because ulnar head excision was performed in all cases to remove inflammatory tissue from the distal radioulnar joint and ease wrist pain. In this context, the most recent follow-up did not reveal ulnar impingement.

Our study showed that the 3DMT-Wrist system can offer the following ROM: palmar flexion by 44.9° ± 6.5°, dorsal extension by 25.8° ± 3.3°, ulnar deviation by 20.2° ± 4.3°, radial deviation by 16.2° ± 3.1°, pronation by 81.6° ± 3.0°, and supination by 80.8° ± 3.5°. Brumfield et al. (Brumfield and Champoux, 1984) found that patients with a wrist joint ROM of 10° palmar flexion and 35° dorsal extension were capable of performing most daily activities. Palmer et al. (Palmer et al., 1985) measured functional wrist motion in 10 normal individuals using triaxial electrical goniometry. They assessed wrist motion by asking the participants to perform 52 standardized tasks; the results showed that normal wrist ROM included 5° flexion, 30° dorsal extension, 10° radial deviation, and 15° ulnar deviation. In this context, a recent study showed that daily hand activities can be accomplished at 60% of the maximum ROM of the wrist (Nadeem et al., 2022). This indicates that the ROM offered by the 3DMT-Wrist system is adequate for all requirements of daily life.

Implant loosening is evaluated by continuous radiographs, and is characterized by permeability around the stem or migration by at least 2 mm from the stem profile to the implanted tantalum bead. Data from a study that performed 10-year follow-up after placement of fourth-generation total wrist prostheses (Table 4) have indicated the overall cumulative implant survival rate (with revision as the primary outcome) to be 92%. On including radiologically loosened implants that had not been fixed, the overall implant survival rate was found to be 75% (Fischer et al., 2020). Five-year survival rates of up to 90%–97% have been observed with Maestro or ReMotion implants. In this context, Biax and Maestro implants have been withdrawn from the market as they are no longer economically viable and the Universal II has been updated to the Freedom version (Harlingen et al., 2011). In a study, the cumulative implant survival for Biax, Remotion, and Maestro implants was found to be 81%, 94%, and 95%, respectively, after 5 years of follow-up; the corresponding rates of radiographic loosening were 26%, 18%, and 2%, respectively (Sagerfors et al., 2015).

**TABLE 4:** Reported results and survival with the cementless fourth-generation TWA implants.

Outcomes	Remotion	Universal 2	Remotion	Universal 2	Motec wrist	New total wrist arthroplasty
Number	69	69	87	95	57	20
Mean follow-up (years)	10	9	7	4.4	8	5.7
ROM	Flexion 0 (−10 to 10) Extension (−10 to 10) Radial Deviation 5 (−10–15) Ulnar Deviation 0 (−5–10) Pronation 0 (−5 to 5) Supination 0 (−10 to 10) Results are shown as median (interquartile range).	Pre-operation: 34° flexion and 36° extension Post-operation:37°flexion and 29°extension	Flexion0 (−10 to 10) Extension 5 (−5–15) Radial deviation 0 (−5 to5) Ulnar deviation 0 (−5–10) Pronation0 (−5 to 5) Supination 5 (−5–10) Results are shown as median (interquartile range).	Dorsiflexion 29 palmar flexion 21	AROM 97–126 Supination 81-83; Pronation 82 -83	Flexion-extension: 42.3
VAS	NA	NA	-2.0 Postoperation minus preoperation	8.1 to 5.4	34 to 8	57.9 to 4.5
DASH						
Pre-operative	NA	NA	NA	61	39	NA
Post-operative	NA	NA	NA	46	25	61.2 Post-operative 1.5year
PRWHE^a^	-35.5	NA	-37.5	NA	NA	NA
Complications	Radial component polyethylene	NA	Loosening of the prosthesis	Persistent wrist pain; joint stiffness	Loose distal component, infection, misalignment	Loose carpal components
Kaplan-Meier cumulative survival (%)	94%	78%	99%	91%	86%	NA
Grip strength (Kg)						
Pre-operative	NA	NA	Statistically significant difference between postoperative and preoperative	4.8	21	NA
Post-operative	6	NA		10	24	NA
Principal Investigator	Fischer et al. https://doi.org/10.1016/j.jhsa. 2020.02.006	Gil JA et al. https://doi.org/10.1007%2Fs11999-017-5445-z	Sagerfors et al. https://doi.org/10.1016/j.jhsa. 2015.09.016 Badge	Badge et al. https://doi.org/10.1302/0301-620x.98b12.37121	Reigstad et al. https://doi.org/10.1016/j.jhsa. 2017.06.097	Ward et al. https://doi.org/10.2106/jbjs.h.01614

^a^ PRWHE, score was analyzing by Postoperative minus preoperative.

The ReMotion system includes a titanium plasma-coated CoCr stem for press-fit fixation in the cranial and radial bones, two CoCr carpal screws, and an ovoid CoCr metal-ultra high molecular weight polyethylene joint (Herzberg et al., 2012). The Motec prosthesis has a sandblasted calcium phosphate-coated titanium stem for cementless screw fixation of the capitate/third metacarpal and radius and a metal-on-metal ball-and-socket joint made of CoCr; a small amount of bone cement is required for TWA (Thillemann et al., 2016). In their study, Holm-Glad et al. (Holm-Glad et al., 2022) observed statistically significant increases in blood Cr and Co levels in participants who had undergone TWA with the metal-on-metal Motec prosthesis. Notably, the 3DMT-Wrist system consists of Ti6Al4V carpal and radial components and a polyethylene joint ball; the radial component consists of a Ti6Al4V shank and a CoCrMo articular socket. The surfaces of the carpal and radial stems have a microporous structure measuring 1 mm in thickness; as they are synthesized in one piece by metal 3D printing, there are no issues related to the loss of coating. In addition, the joint interface of the 3DMT-Wrist system comprises metal-on-polyethylene; metal debris should not therefore be generated during activity.

The 3DMT-Wrist system appeared to offer good clinical results. This may be attributed to the personalization of the implant; the osteotomy position and center of rotation of the native joint were planned individually prior to surgery with the aim of maximal restoration of the biomechanical characteristics of the wrist. In addition, the shape of the osteotomy surface was consistent with that of the radius; this minimized the pressure of the prosthesis on the osteotomy plane. The congruence was maintained with the aim of reducing complications such as prosthesis loosening and sinking. Compared to the fourth generation Motec prosthesis, the 3DMT-Wrist radial component is better shaped to match the inner wall of the cortical bone; this improves stability and strength of the radial component until adequate bone growth occurs. This also prevents early loosening due to differences in elastic modulus between the prosthesis and cancellous bone.

Our study has a number of limitations. First, it had a small sample size. In this context, the 3DMT-Wrist system is newly designed, and is not related to the four generations of total wrist joint prostheses currently in use; the duration of follow-up was therefore limited in addition to the sample size. However, follow-up will be continued and further cases will be included to increase the sample size and validate the results of this study. In this context, Krukhaug et al. found no difference in TWA outcomes between high- and low-volume centers in Norway (Krukhaug et al., 2011). Second, the current study did not to compare outcomes of the novel system with those in current use; this is mainly because TWA has recently gained popularity in China and no available total wrist prostheses have been designed and manufactured in the country. Third, the duration of follow-up was inadequate and no cases required revision; however, the patients will be continuously followed-up in future.

In conclusion, the novel 3D-printed microporous titanium artificial wrist joint prosthesis can significantly relieve wrist pain and improve function with good fixation and ROM. The developed prosthesis demonstrated no dislocation or need for revision after 1.6 years’ follow up; this suggests that the novel design has considerable potential for clinical application.

## Data Availability

The original contributions presented in the study are included in the article/supplementary material, further inquiries can be directed to the corresponding authors.
